# A brief perspective on neural cell therapy

**DOI:** 10.1186/2052-8426-2-2

**Published:** 2014-01-08

**Authors:** Jan Pruszak

**Affiliations:** Emmy Noether-Group for Stem Cell Biology, Institute of Anatomy and Cell Biology, University of Freiburg, Albertstr. 17, 79104 Freiburg, Germany

**Keywords:** Cell therapy, Neural stem cells, Neural transplantation, Neurological disease

## Abstract

For a range of nervous system disorders current treatment options remain limited. Focusing on Parkinson’s disease as a neurodegenerative entity that affects an increasing quantity of people in our aging societies, we briefly discuss remaining challenges and opportunities that neural stem cell therapy might be able to offer. Providing a snapshot of neural transplantation paradigms, we contemplate possible imminent translational scenarios and discuss critical requirements to be considered before clinical implementation.

## Background

**Figure 1:**
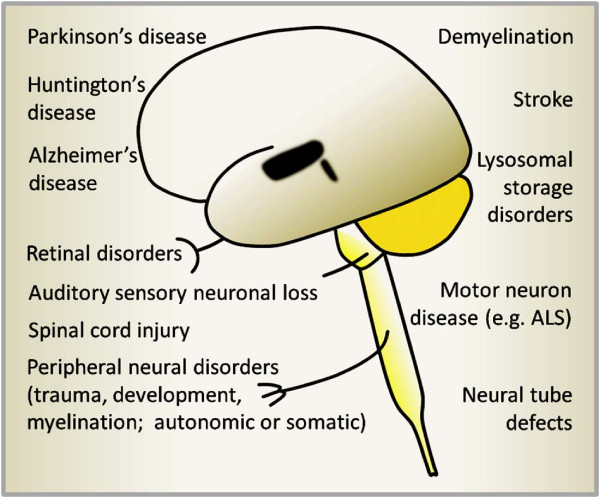
**Candidates for neural cell replacement therapy?** A range of neurological disorders affecting the central as well as the peripheral nervous system are being considered to be amenable to cell therapeutic intervention. These include defects associated with aging, injury and/or genetic and developmental disorders. Neural cell sources to be administered include neural stem and precursor cells, astro- or oligodendroglial preparations and postmitotic neurons.

Repairing the central nervous system may appear daunting in light of how little we still understand about its intricate molecular and cellular structure. Nevertheless, concepts of neural cell transplantation were explored early on, and since the 1980s there have been more concentrated systematic efforts in the area of Parkinson’s disease, specifically, which is used here as a salient example (for review, see [1]) (Figure 1). Its progressive, widespread pathology includes a rather circumscribed epicenter of cell loss: the dopaminergic neurons located in the ventral midbrain which send axonal projections to target cells in the striatum and play a critical role in the control of voluntary movement. Based on a solid fundament of rodent and primate studies, functional replacement of the dopaminergic neuronal subset by engrafting fetal midbrain tissue has been unequivocally demonstrated (for review, see [2, 3]).

## Discussion

**Figure 2:**
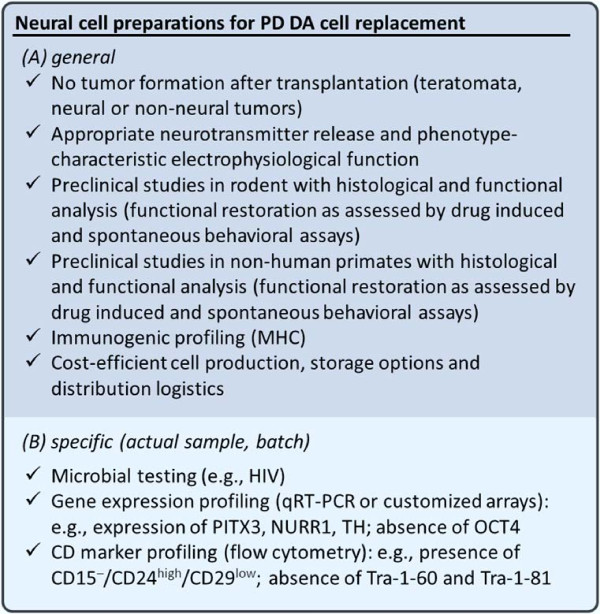
**Score card for cell preparations for clinical transplantation in Parkinson’s disease.** Parameters listed here may serve to illustrate criteria that would need to be met by a cell source before application as a therapeutic agent to treat neurological disease. **(A)** *General* features refer to properties to be ensured in a range of pre-clinical, biotechnical studies regarding the cell source in question. This includes safety-related (e.g., absence of tumor formation) and also efficacy-related issues such as phenotypic functionality (e.g., histological integration into the host tissue and functional restoration of the neural circuitry). **(B)** *Specific* features refer to properties that would be considered as a means of quality control of each batch or cell preparation in clinical-biotechnological routines as part of the actual clinical trial. Examples of intracellular or surface markers that may be used to characterize (dopaminergic) neuronal cell preparations are given.

This body of work has led to clinical trials (for review and meta-analysis, see [4]) including double blind, sham-surgery controlled [5, 6] and open-label studies with varying clinical and neuropathological outcomes and assessments [7–9]. The important proof-of-principle, however, that neural cell preparations *can* survive long-term, restore function and alleviate neurological disease by means of “replacement” was provided by these studies [10, 11]. In addition, cell preparations from various tissues of origin have been used in neural cell therapeutic paradigms, including long term-expanded neural lines, hematopoietic and other mesenchymal-derived sources. The associated disease modulatory effects may carry the potential for medical benefit, and non-neural cells could serve as vehicles for trophic factor release or gene delivery but do not represent cell replacement in the sense of the original ambitions. A number of phase-1 clinical trials have been initiated based on, at least partially, comparably limited sets of pre-clinical data. When considering such paradigms, one critical demand will be that the overall efficacy supersedes that of current standard or alternative experimental therapeutic options (even if the threshold to clinical translation appears somewhat lower due to the perceived “safer” character of such sources [12, 13]). In addition to continuously improving pharmacological therapies (for review, see [14]), as well as neurostimulatory approaches using electrode implantation (deep brain stimulation) (for review, see [15]), RNA interference-based approaches that aim for amelioration of disease by preventing formation of the pathognomonic neural protein aggregates, for instance, have to be considered as potentially feasible, promising therapeutic alternatives (for review, see [16]). Still, the lasting structural and functional repair of neural circuits by means of cell replacement is a worthy goal with potential biomedical benefit beyond what experimental alternatives may offer [17]. To achieve more reliable and predictable outcomes post-transplantation of fetal ventral midbrain tissue for Parkinson’s disease, an ongoing multicenter clinical program aims at fine-tuning and coordinating neural transplantation procedures and protocols [18]. Long-term it is unlikely, as ethically problematic and technically hardly feasible, to consider human fetal neural cell preparations for future clinical routines. What are some concrete milestones to achieve in bringing neural cell replacement to the clinics? Among the most important requirements is the generation of the actual therapeutic agent, i.e. the cell preparation to be administered, and pluripotent sources may provide a scalable and potentially efficacious alternative [19, 20]. It will be critical to specify functional equivalents of the phenotypes in need, and to exclude uncontrolled generation of proliferative or otherwise unwanted subtypes [21, 22]. On a cellular level, this requires the reliable control of gene regulatory networks that define the phenotype of interest [23], on a biotechnological level the development of reliable, scalable protocols that predictably generate the desired cell type from pluripotent or multipotent sources [24]. Learning from the ontogenic neural stem cell niche, extracellular matrix molecules, growth and patterning factors or small molecule inducers can be applied to generate cells that approximate the physiological equivalent *in vitro* (for review, see [25]). A cell source to be applied in a therapeutic context requires an explicit “score card” of characteristics that need to be met (Figure 2), which may include expression of definitive gene loci, transmitter release and electrophysiological activities and surface molecular patterns. The first and the latter can be economically and efficiently tested by implementing customized arrays [26] and flow cytometric readouts [21, 22] into the cell production routines. Although the central nervous system has been regarded as a somewhat immune privileged site [27], the immunogenic potential of the cell preparation also needs to be considered [28]. Patient-derived induced-pluripotent stem cell preparations provide options for autologous transplantation, but it remains to be seen whether the complex methodological work-flows (donor cell harvesting, reprogramming, expansion, induction of phenotype, transplantation) could ever be sufficiently economized to make this a clinical reality. Easier to envision is a cell bank with major histocompatibility complex profiles that closely match particular patient subsets [29]. Of equal significance to basic research and biotechnological efforts is the parallel refinement of medical parameters which include the identification of:

- The best candidate disease, the most appropriate patient collective and disease stage to intervene, taking into account the conditions of the host/ recipient tissue at the implantation site.
- The most appropriate means of delivery and diagnostically conclusive readout assessments.
- The means to avoid potential unwanted side-effects and to ensure proper access to follow-up and parallel supportive therapies.

In a nascent journal on Molecular and Cellular Therapies, one may explore opportunities for further innovative developments. Induced pluripotent stem cell technology [20] and direct phenotype conversion by epigenetic modifications (iN and iNS cells; for review, see [30]) paired with novel tools for genome editing of human cell lines (for review, see [31]) make it conceivable that disease correction of such autologous cell sources could efficiently be performed before readministration to the patient. Epigenetic (re)programming will require sophisticated insights into the most appropriate gene expression make-up (dosage and ratios) of the needed phenotype. Moreover, continuous optimization of directed cell differentiation and recent developments in 3D differentiation systems that imitate embryological tissue context may provide an avenue to yield close-to physiological cell types [32]. In terms of delivery, cell administration via the blood stream or cerebrospinal fluid (intrathecally) may eventually complement the current standard neuro-stereotactic approaches. Potentially, one could exploit certain “cellular homing” behavior to a lesion in certain contexts (tumors, inflammation, ischemia). For neurodevelopmental disorders, modes of delivery may include *in utero* surgery (for review of spina bifida as a clinical example, see [33]). Finally, an attractive option is to enhance the limited neuroregenerative capacities of the brain itself for *intrinsic* cell replacement (for review see [34]). As illustrated by the rapid and unexpected developments in stem cell biology of the past few years and months, predicting the actual biomedical realities may be difficult. More important may be postulating self-imposed, if not regulatory, criteria that could guide future developments:

- To remain aware of the considerable responsibilities that come with conducting work (funded) to diminish human suffering caused by disease and the concomitant hopes put into our daily work.
- To not compromise on the demand for functional effects and to critically evaluate the need for animal studies before clinical translation (behavioral recovery).
- To educate the public, including patients, caretakers and family members as well as organizations (and funding bodies) and moderate their enthusiasm, hope and unjustified expectations, where appropriate.
- To remain cautious about too early clinical translation and to take clear stands against clinical practice not sufficiently founded on scientific evidence (see [12, 13]).

Adherence to such principles ensures lasting trust toward this field and may avert unwarranted hype, while maintaining the justifiable enthusiasm and motivation that clinicians, physician-scientists and basic researchers working in the fields of regenerative medicine and applied stem cell biology share in terms of bringing the joint scientific efforts to clinical fruition.

## Conclusion

As optimized protocols generate increasingly authentic and safer neural cell preparations, future efforts may focus on refining parameters of patient selection and cell delivery. Ultimately, enhanced interdisciplinary dialogue and applying the highest scientific standards on all levels from basic stem cell biology to clinical protocol development may make neural cell restoration via cell therapy a clinical reality.
